# Francisco José de Goya y Lucientes (1746–1828). Self-portrait with Doctor Arrieta (1820).

**DOI:** 10.3201/eid1005.AC1005

**Published:** 2004-05

**Authors:** Polyxeni Potter

**Affiliations:** * Centers for Disease Control and Prevention, Atlanta, Georgia, USA

**Figure:**
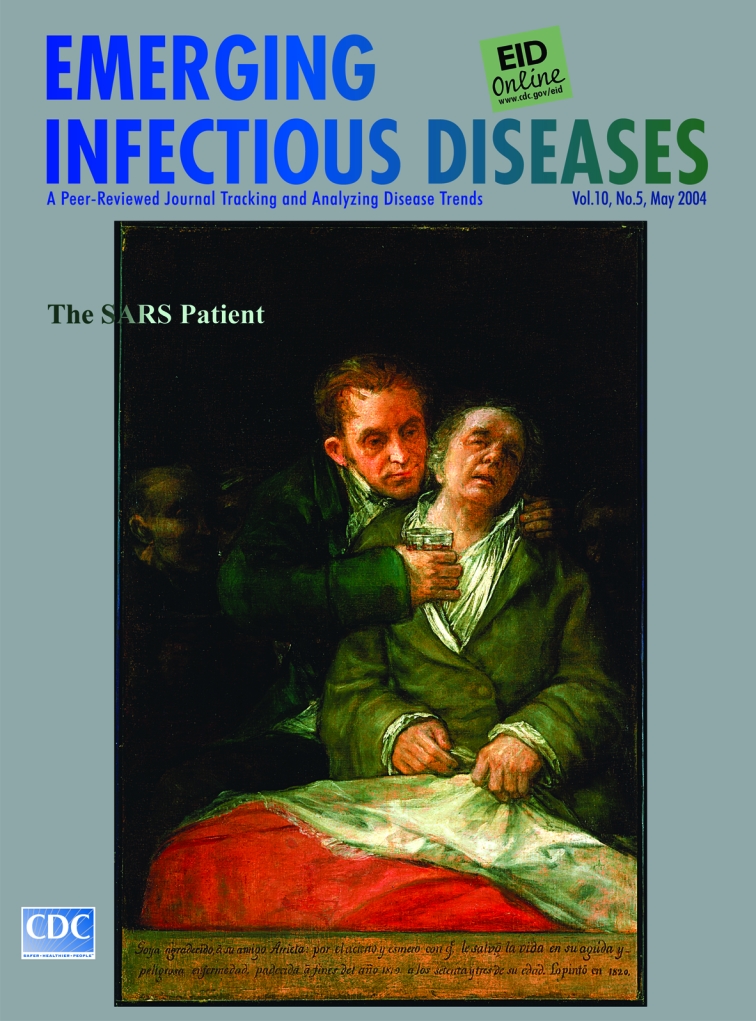
Francisco José de Goya y Lucientes (1746–1828). Self-portrait with Doctor Arrieta (1820). Oil on Canvas (114.62 x 99.38 x 9.53 cm). The Minneapolis Institute of Arts, The Ethel Morrison Van Derlip Fund Cover topic: The SARS patient

“Goya in gratitude to his friend Arrieta for the skill and care with which he saved his life in his acute and dangerous illness suffered at the end of the year 1819 at the age of 73. He painted it in 1820” (*1*) reads the inscription at the bottom of Goya’s self-portrait on this month’s cover of Emerging Infectious Diseases. An affirmation of medical practice, the painting is also an acknowledgment of human compassion, a quality the artist thought extremely rare.

Conflicted in his acceptance of the world and in his portrayal of it and deeply mistrustful of human nature, Goya lingered on the dark side as he painted the full spectrum of life experiences (*2*,*3*). During his long artistic career, he dwelled on the tensions of Spanish society of his day, whose institutions, including medicine, he gleefully satirized (e.g., Of What Illness Will He Die?) (*4*).

Deaths in his family and debilitating illness throughout his years often interfered with his work and left him weak and disillusioned. “Neither sight, nor pen nor inkwell; all these I lack and all that is plentiful is my will,” the painter remarked to a friend regarding his loss of hearing, poor health, and frail disposition (*2*). Near the end of his life, once again he became seriously ill. Overcoming his natural aversion to authority, he entrusted himself to the care of a physician friend. When the health crisis subsided, Goya created Self-portrait with Doctor Arrieta.

Unlike most paintings of his later years, which evoke horror and darkness, this double portrait imprints a gentle aspect of humanity on the mild physiognomies of physician and patient. Even so, rather than a departure from his sinister worldview, the painting of one man tending to another was a gesture of gratitude after deliverance from death (*5*).

The portrait is an empathetic rendition not of Goya alone but of the universal human patient. Isolated but for the intruding shadows witnessing his pending demise, in a drab dressing gown, generic, exposed, and vulnerable, Goya embodies the plight of the sick. Withered and limp, unkempt and undignified, he is reduced to an infantile state, to be comforted and cajoled, humored with therapeutic potions and measures, and ordered to obey.

Gone is the thundering presence, the compelling personality, the artistic genius, the signature mistrust of human nature. Opinions and attitudes were shed at the sickroom door, along with his everyday clothes and his ability to walk and control his life. With his private condition on public display, he is at the mercy of his caretakers. Clutching the carmine blanket between him and the world, he succumbs to the physician’s sympathetic embrace and, near death, sinks deeper into isolation.

The kindly physician is warm and obliging if not unduly hopeful. Aware of his limited capacity to reverse the course of illness, he focuses on what is within his capacity, comfort and support. He draws near the patient, as if to become one with him and propel his own strength and energy onto the ailing body. The closeness of his embrace equals his instinct to alleviate pain and his oblivion of risk to himself from proximity to the patient. As he firmly administers the medication, his face wears the look of the stoic philosopher and the eagerness of the medical intern.

An astute observer of the human condition, Goya understood the tragic nature of disease, often manifested in our inability to prevent its onset, control its course, and predict its outcome. Understanding of infection has burgeoned since 1820, yet patient isolation, vulnerability, uncertainty, and untimely death remain unresolved. In emerging disease puzzles, where treatment is sometimes administered while large pieces are still being assembled, the old measures of infection control and quarantine are challenged by new environmental, social, and scientific developments. Contagion, unknown to Dr. Arrieta, is particularly pertinent in diseases like SARS (*6*), where the threat is not fully quantified until, unlike the images in Goya’s double portrait, the patient and the caretaker are one.
